# An innovative approach to predicting global warming without using climate model simulations

**DOI:** 10.1093/nsr/nwae453

**Published:** 2024-12-12

**Authors:** Guang J Zhang

**Affiliations:** Scripps Institution of Oceanography, La Jolla, CA, USA

Global warming projections rely heavily on climate model simulations. Due to too many tuning knobs in climate models, such as the representation of clouds and convection, global warming projections have a large range of inter-model spreads. Climate sensitivity, which measures how much the Earth's surface temperature increases when the amount of CO_2_ in the atmosphere is doubled in a future climate, strongly depends on the feedback from clouds and convection, albedo, water vapor and atmospheric temperature, etc. The climate sensitivity and related climate feedbacks have been studied extensively in the literature. Yet, their uncertainties remain high [1]. A major stumbling block is the uncertainty in non-temperature feedbacks such as cloud feedback.

In the conventional partial radiative perturbation (PRP) approach, temperature change due to external forcing such as that from doubling CO_2_ is estimated by $\delta {{T}_s} = - F/\\lambda $, where *F* is the external radiative forcing, $\\lambda = \sum {{\\lambda }_i}$ is the sum of temperature feedback and non-temperature feedbacks from water vapor, clouds, and surface albedo [2–4]. The individual non-temperature feedback component ${{\\lambda }_i}$ can be either positive or negative. As the feedback parameters are in the denominator, the sum of both positive and negative feedback values can become a small net value. As such, even a small uncertainty in the feedback estimate of an individual component can lead to large uncertainties in estimated climate sensitivity.

The analysis framework proposed in [5] departs drastically from the conventional PRP approach that uses radiative kernels, with two major advances. One is the introduction of energy gain kernel (EGK) for temperature feedback and the other is the non-temperature feedback kernel at the surface A_NT_. The EGK for temperature feedback amplifies the energy perturbations from both external forcing and internal non-temperature feedback processes. Its discovery rectifies the common misconception in existing radiative kernels in climate feedback studies that portray temperature feedback as predominantly negative. The physical interpretation of EGK as an amplifier of the external forcing is superior to the PRP approach.

Relating non-temperature feedback kernel at the surface, A_NT_, to the mean climate information, is a brilliant idea. It sidesteps the need to use global climate model simulations to estimate non-temperature feedbacks. A_NT_ utilizes the amplification of the net solar energy absorbed by the surface through downward thermal radiation from the atmosphere in the climate mean state for estimating the amplification of external energy perturbations at the surface by non-temperature feedback in the perturbed climate state. Cai *et al.* [5] show that the spatial pattern of this amplification in the mean climate closely resembles the spatial pattern of the amplification of CO_2_-induced external energy perturbations at the surface by non-temperature feedback in the perturbed climate state simulated by climate models.

The innovative concepts of EGK and non-temperature feedback kernel at the surface A_NT_ allow the authors to predict global warming by accounting for the amplification of CO_2_-induced external energy perturbations at the surface by both temperature and non-temperature feedback without using climate model simulations.

Despite the scientific advance of the theoretical analysis framework and the robust prediction of global mean warming, there are notable limitations to the work of [5]. The use of the spatial pattern of the greenhouse effect in the mean climate to infer the spatial pattern of the amplification of external surface energy perturbations by non-temperature feedback is an analogue approach. It would not work well when the external surface energy perturbations do not exhibit a similar spatial pattern to the net solar energy absorbed by the surface in the mean climate. For example, this approach would fail to predict surface temperature response to anthropogenic aerosol forcing, as it is relatively highly localized. This may be the main reason why their prediction does not capture the oceanic response to CO_2_-induced forcing because oceanic response has distinctly different spatial patterns from that of the net solar energy absorbed by the surface in the mean climate. This obviously should be the target for further refinement of their work in the future.

***Conflict of interest statement.*** None declared.
